# Immunotherapeutic Strategies for Canine Lymphoma: Changing the Odds Against Non-Hodgkin Lymphoma

**DOI:** 10.3389/fvets.2021.621758

**Published:** 2021-08-26

**Authors:** Joana N. R. Dias, Ana S. André, Sandra I. Aguiar, Solange Gil, Luís Tavares, Frederico Aires-da-Silva

**Affiliations:** Centro de Investigação Interdisciplinar em Sanidade Animal, Faculdade de Medicina Veterinária, Universidade de Lisboa, Avenida da Universidade Técnica, Lisbon, Portugal

**Keywords:** cancer, comparative oncology, non-Hodgkin lymphoma, canine lymphoma, cancer immunotherapy

## Abstract

The new era of immune-oncology has brought complexities and challenges that emphasize the need to identify new strategies and models to develop successful and cost-effective therapies. The inclusion of a canine model in the drug development of cancer immunotherapies is being widely recognized as a valid solution to overcome several hurdles associated with conventional preclinical models. Driven by the success of immunotherapies in the treatment of human non-Hodgkin lymphoma (NHL) and by the remarkable similarities of canine NHL to its human counterpart, canine NHL has been one of the main focus of comparative research. Under the present review, we summarize a general overview of the challenges and prospects of today's cancer immunotherapies and the role that comparative medicine might play in solving the limitations brought by this rapidly expanding field. The state of art of both human and canine NHL and the rationale behind the use of the canine model to bridge the translational gap between murine preclinical studies and human clinical trials are addressed. Finally, a review of currently available immunotherapies for canine NHL is described, highlighting the potential of these therapeutic options.

## Graphical Abstract

The application of canine lymphoma as an animal model for immunotherapeutic approaches in comparative medicine provides an integrated drug discovery platform that maximize interdisciplinary cooperation and leverage commonalities across humans and dogs for the development of novel immunotherapies against non-Hodgkin lymphoma, benefiting both species. 
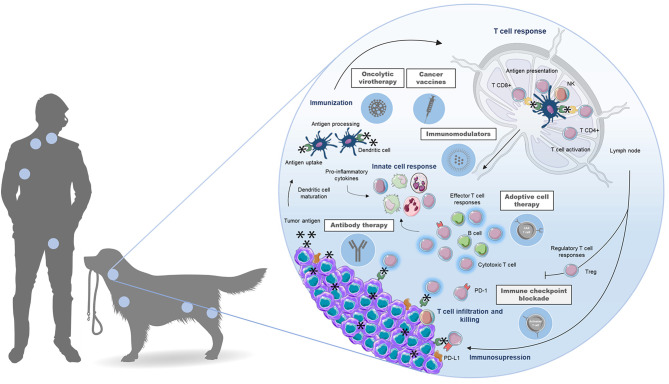

## Introduction

In 2018 alone, cancer was responsible for an estimated 9.6 million deaths worldwide in countries of all income levels, ranking second place in the leading causes of death, behind cardiovascular diseases (1). Owing to population growth, aging, and adoption of lifestyle behaviors associated with cancer risk, this number is expected to rise by about 70% over the next 20 years (2, 3). Still, even though these impressive numbers demonstrate that cancer burden remains a major challenge worldwide, recent developments in personalized medicine and novel treatment approaches, such as immunotherapy, have raised hope of significantly improving cancer survival (2).

The concept of harnessing the host's immune system to treat cancer can be traced back decades, however only in recent years immunotherapies have emerged as a clinically validated and effective treatment strategy (4). Nowadays, cancer immunotherapy has emerged as a fast-growing field and rapidly became the fourth pillar of cancer care, along with surgery, cytotoxic therapy and radiotherapy (5). More recently the successes of clinical breakthroughs, such as checkpoint inhibitors and engineered T cells, revitalized the field and highlighted the opportunities that immunotherapeutic approaches can offer, which culminated in the nomination of “cancer immunotherapy” as 2013's Breakthrough of the Year by *Science* (6, 7). In 2018, the Nobel Prize in Physiology or Medicine was jointly awarded to James Allison (University of Texas MD Anderson Cancer Center) and Tasuku Honjo (Kyoto University School of Medicine) for their discoveries leading to new approaches in harnessing the immune system to fight cancer (8–12).

However, by transforming the cancer therapeutic landscape, this complex modality brought unique challenges to the drug discovery community. In fact, as more cancer patients have received immunotherapies, some of the major drawbacks of these treatments have become clear. One of the major issues is to determine the sub-populations of patients who will respond and who will experience significant toxicities (13). In fact, the challenge now is to extend the range of patients that benefit from immunotherapy while minimizing treatment-related adverse events. To address this, it is crucial to identify factors predictive of response that may help to properly select patients for treatment, identify rational combination therapies, and define progression and resistance (14). This is particularly critical when developing cancer immunotherapies, considering that the patient's immune system is expected to be as significant as tumor-related aspects when determining response and toxicity (15).

Clinical translation of cancer immunotherapy depends on preclinical investigation and rodent models have been the foundation of preliminary basic investigation and safety assays (16). However, these models underrepresent the heterogeneity and complex interaction between the human immune cells and cancers. Indeed, laboratory mice rarely develop spontaneous tumors, are housed under specific-pathogen free conditions that greatly impact immune development, and incompletely model main characteristics of the tumor/immune microenvironment, creating challenges for clinical translation. As a result, these murine models have failed to correlate with clinical success rates, demonstrating an urgent need for innovative pre-clinical models (17–19). Thereby, the use of alternative animal models is pivotal to bridge the translational gap between murine models and human clinical studies. In particular, preclinical models displaying intact immune systems that closely resemble the human immune response, present comparable, spontaneous oncogenesis and immune interactions similar to humans, and can model key clinical outcomes such as efficacy, dose response, and toxicity, will be critical for translational cancer immunotherapy research (15).

Thus, comparative medicine offers an important platform with innovative complex cross-species models that allow the research of novel therapeutic strategies and agents for diseases that are common to animals and humans (20, 21). Notably, the canine model represents a powerful resource of models for cancer immunotherapy research. Dogs are an appealing outbred combination of companion animals that experience spontaneous cancer development in the setting of an intact immune system (15). Besides, naturally occurring tumors in dogs present many clinical, pathological, immunologic, molecular, diagnostic and therapeutic similarities to those observed in humans, that are difficult to reproduce in other models (22–25). This allows studying the complex immune interactions during the course of treatment while also addressing long-term efficacy and toxicity of cancer immunotherapies (15).

Nevertheless, the integration of the canine model in immunotherapeutic approaches research requires diagnosis, staging and treatment response assessment, optimization and standardization, to perform large and organized clinical trials and to achieve conformity when analyzing data (26).

Driven by the great success accomplished with the application of immunotherapies in the treatment of human non-Hodgkin (hNHL) and by the remarkable similarities of canine non-Hodgkin lymphoma (cNHL) to its human counterpart, cNHL has been one of the main focus of comparative research regarding the development of immunotherapeutic approaches for dogs (**Graphical abstract**).

## Rationale for a Canine Model of Lymphoma

For a long time, research in lymphoma has benefited from traditional mouse models, however the paucity of truly representative models has hindered complete understanding of disease biology and drug development. With the introduction of genomics technology, non-traditional animal models have been more accessible and the leverage of these opportunities may represent a novel strategy to accelerate disease research and new drug discovery (27). Furthermore, there is an increasing number of studies demonstrating that spontaneously arising lymphoma in dogs could be an invaluable resource to study the biology and treatment of this disease (28). As such, the cNHL model may help to bypass many of the limitations associated with the use of murine models while presenting other additional advantages (29, 30).

The cNHL shares many remarkable similarities with its human counterpart (29, 31–34). The incidence of cNHL of 15–30/100 000 is similar to human incidence (35, 36), though additional studies indicate that the incidence of cNHL may be higher (37). Classification and grading schemes of cNHL were designed to reflect the equivalent in people and facilitate comparison. In fact, the 2008 revised World Health Organization classification based on the Revised European American Lymphoma classification system, which attempts to group lymphomas by cell type, phenotypic, genetic and molecular aspects, is the current standard for the diagnosis and classification of human lymphoma, also serves as the basis for the current canine recommendations (38, 39). The use of these current World Health Organization guidelines as a template, allowed describing 20 cNHL entities, among nearly 50 discrete subtypes of hNHL. Moreover, B-cell lymphoma is more prevalent than T-cell lymphoma in both species and diffuse large B-cell lymphoma is the most common type of non-Hodgkin lymphoma (NHL) in both humans and dogs (38). Finally, treatment modalities for cNHL are similar to those used for human lymphoma (radiation, corticosteroids, chemotherapy) and CHOP (cyclophosphamide, doxorubicin, vincristine, and prednisolone)-based chemotherapy agents are typically used to treat it. Response to treatment and resistance also present clinical patterns similar to hNHL (27).

From a drug development perspective, the canine model represents a large and long-lived animal model, evolutionarily more closely related to humans than rodents, that provides a more accurate assessment of the pharmacokinetic/pharmacodynamic parameters, while determining safety and efficacy of new therapeutic agents and approaches (27, 40). Moreover, the relatively fast disease progression rate allows obtaining early conclusions from clinical trials. In fact, a randomized clinical trial in pet dogs requires ~1–3 years, whereas a human clinical trial takes about 15 years to be completed. This short timeline allows to integrate the findings of pet trials on human trials, including toxicity, response, pharmacodynamics, dose, regimen, schedule, biomarkers and responding histology assessment (28).

Another main advantage of the canine model is that cNHL is a spontaneously occurring tumor in an immune-competent host, in contrast to murine xenograft or genetically engineered mouse models. This natural occurring cancer setting offers genetic diversity similar to human lymphoma and allows studying biological mechanisms, such as tumor initiation and promotion. Moreover, the pet dog model harnessed by the evolutionary conservation allows to identify similarities between canine and human lymphomagenesis, for example in identifying key “driver” gene mutations common to both species (27).

The benefits of the cNHL model extend beyond the biological advantages of a spontaneously occurring tumor in a large animal. Pet dogs share the same living environment as their caregivers, allowing to study environmental risk factors of developing lymphoma (27, 28). For example, an epidemiological study in France demonstrated a correlation between the incidence of cNHL and hNHL and reported a strong association between cNHL and the distribution of waste incinerators, radioactive waste or other polluted sites (41). Moreover, there is an increased prevalence of lymphoma within specific dog breeds (42) and a breed-specific distribution of B-cell and T-cell lymphomas (43). This in association with the well-organized multi-generational pedigrees kept by many breeders, represents a unique genetic advantage that allows mapping of lymphoma predisposition genes with strategies that are not possible in humans (28).

The final rationale for using dogs with lymphoma as an animal model relies on the dual benefit concept of this comparative research approach. Improved current health care have promoted the increase of dogs lifespan, allowing the diagnosis of late-in-life diseases such as cancer (44). Lymphoma particularly is one of the most common malignancies in dogs (28). In addition, the social status of dogs as companion animals allows them to benefit from high quality health care and the ethical exploration of translational approaches. Moreover, these initiatives are also motivated by the increasing healthcare standards demanded by pet owners, creating a need for novel cancer therapies in veterinary settings (20, 21, 45). Altogether, the use of the cNHL model represents a unique opportunity to strengthen the collaboration between human and veterinary medicine in lymphoma research, that ultimately will lead to advances in the care of people and dogs affected by NHL, a critical medical unmet need of today's society (22, 27).

## A Critical Unmet Need for Novel Treatment Options for Non-Hodgkin's Lymphoma in Comparative Oncology

NHL, an heterogeneous group of cancers characterized by a diverse class of lymphocyte proliferations, represents one of the most common neoplasias in both humans and pet dogs (38, 46). hNHL constitutes the most commonly reported hematological malignancy worldwide, comprising nearly 3% of all cancer diagnoses. The highest incidence rates are found in Australia/New Zealand, Northern America, and Europe. In the United States, NHL is the seventh most common and sixth most common cause of cancer-related death, in Europe is the eleventh most common and the fourteenth most deadly malignancy and its incidence has nearly doubled since the early 70s (47, 48). NHL represents 90% of all lymphomas and encompasses an heterogeneous group of cancers characterized by the proliferation of malignant lymphocytes, 85–90% of which arise from B lymphocytes, whereas the remaining derive from T cells or natural killer cells. This diverse group of malignancies usually develops in the lymph nodes, but can occur in almost any tissue, ranging from the more indolent follicular lymphoma to the more aggressive diffuse large B-cell (DLBCL) and Burkitt's lymphoma (49). NHL patients typically present with persistent painless lymphadenopathy, but some patients may present with constitutional symptoms or with involvement of organs other than those from the lymphoid and hematopoietic system (50).

The basis of treatment selection requires an accurate diagnosis, a careful staging of the disease, and the identification of adverse prognostic factors. Regardless, NHL patients most commonly receive chemoimmunotherapy as initial treatment. Radiation therapy may be performed if patients have early-stage disease (50). Response rates to conventional chemotherapy are generally >50%; however, most patients eventually relapse. Moreover, the toxicity of conventional chemotherapy often limits its efficacy (47).

In the last decades, the scientific community has been reporting cases of therapeutic success using monoclonal antibodies (mAbs) in the treatment of NHL in humans. One of the most successful examples has been the application of mAbs targeting the surface antigen of CD20 (Rituximab®) in combination with chemotherapy regimen CHOP, which has revolutionized the treatment of B-cell lymphoma by significantly improving disease-free interval and overall survival, with minimal toxicity (51, 52). Even though current therapy strategies have significantly improved prognosis of patients diagnosed with NHL, a substantial fraction of patients relapse or are refractory to these treatments. Several treatment shortcomings have been identified as research priorities, however rituximab resistance and refractory/relapsed disease represent major current and emerging challenges (53–55).

Thus, a plethora of new immunotherapeutic approaches to treat lymphoma have been ensued. The most exciting classes of immunotherapies comprise chimeric antigen receptor T-cells, bispecific antibodies, immune checkpoint inhibitors, and vaccines. The advent of such innovative therapies brought unique challenges that need to be considered, including the assessment of the appropriate timing of treatment, optimal patient population, duration of therapy, toxicity, and cost. Hence, future studies need to focus on the development of new strategies, models and paths in order to optimize the drug development of novel immunotherapies for hNHL (56).

Owing to shared molecular, incidence, genetic, histopathologic and clinical features, cNHL has been proposed as a comparative animal model for the research of novel therapeutic agents and approaches for hNHL (22–24, 30). cNHL displays several histological subtypes and patients can manifest a wide range of symptoms. However, most suffer from generalized lymphadenopathy (multicentric form) and are diagnosed with intermediate to high-grade lymphoma, more commonly of B-cell origin. Without treatment, the disease has high mortality (28), requiring prompt chemotherapy to achieve temporary remission and prolonged survival. Chemotherapy still remains the mainstay for the treatment of cNHL and regardless of the numerous published chemotherapeutic protocols, it seems we have reached a stalemate concerning what this treatment modality has to offer in standard settings (57). Yet, cure is rarely achieved and the majority of dogs relapse with lethal, drug-resistant lymphoma. The 12 month median survival barrier and the 20 to 25% 2 years survival rates demonstrate an urgent and unmet need in veterinary medicine to develop new treatment strategies for refractory disease (58–61).

**Figure 1:**
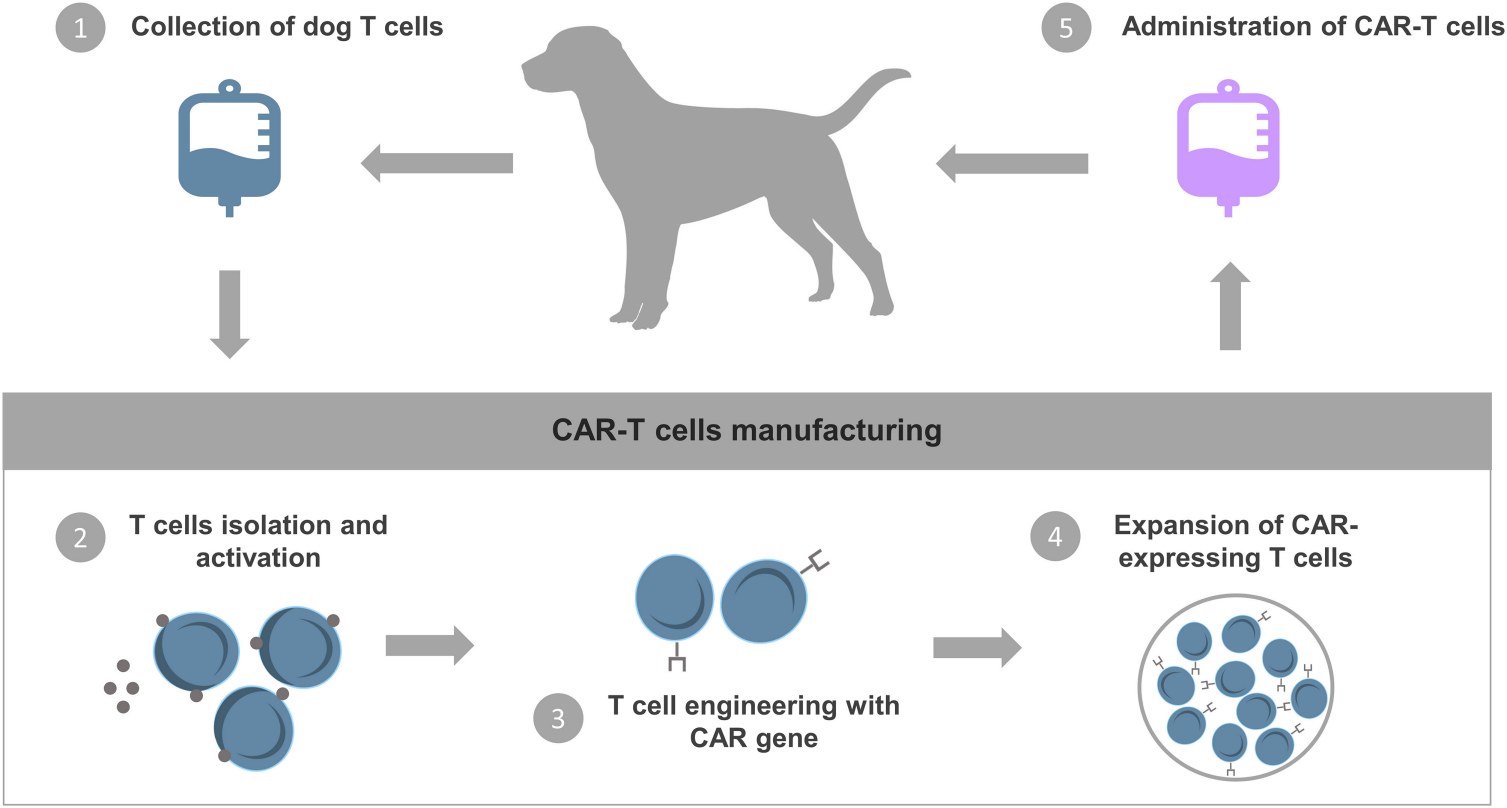
Schematic representation of available and under development immunotherapy strategies for cNHL. Currently, several research groups are actively investigating new immunotherapies that mobilize the patient's own immune system to treat NHL in both pets and pet owners. These treatment modalities include therapeutic mAbs that promote the direct or indirect death of cancer cells, adoptative cell transfer that uses a patient's own cells to induce antitumor activity, oncolytic virotherapy that involves the replication-competent virus in the elimination of cancer, immunomodulators that aim to enhance immune responses and tumor control and vaccines that stimulate a patient's own immune system against cancer cells.

Thus, immunotherapies for cNHL are a promising approach for the development of a new class of anti-cancer therapeutics, which will in many cases benefit humans and man's best friends. To demonstrate the potential of these strategies, available and under development immunotherapies for cNHL will be summarized below (Figure 1).

## Current Immunotherapies for Canine Non-Hodgkin's Lymphoma

After decades of weakening or even eliminating the patient's immune system with chemotherapy, now the trend is to harness the ability of the immune system to eradicate cancer (62). Over the past decades immunotherapy has moved into the forefront of cancer care due to unprecedented clinical success in a wide range of malignancies, sometimes even in late stages of disease (63). The field of veterinary immunotherapy holds similar promise for companion animals with cancer, and several efforts have been made in order to develop veterinary specific immunotherapies (Table 1). In the nearby future, it is hoped that tumor immunotherapy will become a valid therapeutic tool in veterinary oncology, along with chemotherapy, radiotherapy and surgery.

**Table 1:** Immunotherapy approaches developed and under development for cNHL.

**Monoclonal antibodies therapy**	**Study**	**References**
mAb 231	Preclinical and clinical	(64–66)
Anti-HLA-DR (L243)	Preclinical and clinical	(67)
Anti-HLA-DR (IMMU-114)	Preclinical and clinical	(67)
Anti-CD20 (6C8)	Preclinical	(51)
Anti-CD20 (1E4-cIgGB)	Preclinical and clinical	(68)
Anti-CD20 (NCD1.2)	Preclinical	(69)
Anti-CD20 (AT-004)	Preclinical and clinical	Aratana Therapeutics®
Anti-CD52 (AT-005)	Preclinical and clinical	Aratana Therapeutics®
Anti-CD20 (1E4-cIgGB) plus CD47 blockade	Preclinical	(45)
Anti-CD20 (4E1-7-B_f)	Preclinical and clinical	(70)
**Adoptive cell transfer therapy**	**Study**	**References**
Autologous T cells	Preclinical and clinical	(71, 72)
Autologous T-cells		Aurelius BioTherapeutics®
Autologous T-cells plus tumor vaccination		Elias Animal Health®
CD20 CAR-T cells	Preclinical and clinical	(73)
**Oncolytic virotherapy**	**Study**	**References**
Canine distemper virus (pCDVeGFPN)	Preclinical and clinical	(74, 75)
Newcastle disease virus	Preclinical and clinical	(76, 77)
Reovirus (dearing strain)	Preclinical and clinical	(78, 79)
**Immunomodulator therapy**	**Study**	**References**
Autologous tumor antigen-coated microbeads with IL-2 and GM-CSF	Preclinical and clinical	(80)
**Vaccine therapy**	**Study**	**References**
Intralymphatic autologous tumor vaccine	Preclinical and clinical	(81–83)
Autologous CD-40-activated B-cells loaded with total RNA from autologous lymphoma cells	Preclinical and clinical	(84)
DNA-vaccine targeting canine telomerase reverse transcriptase	Preclinical and clinical	(85–87)
Autologous tumor heat shock proteins (APAVAC)	Preclinical and clinical	(88–90)

### Monoclonal Antibodies

**Figure 2:**
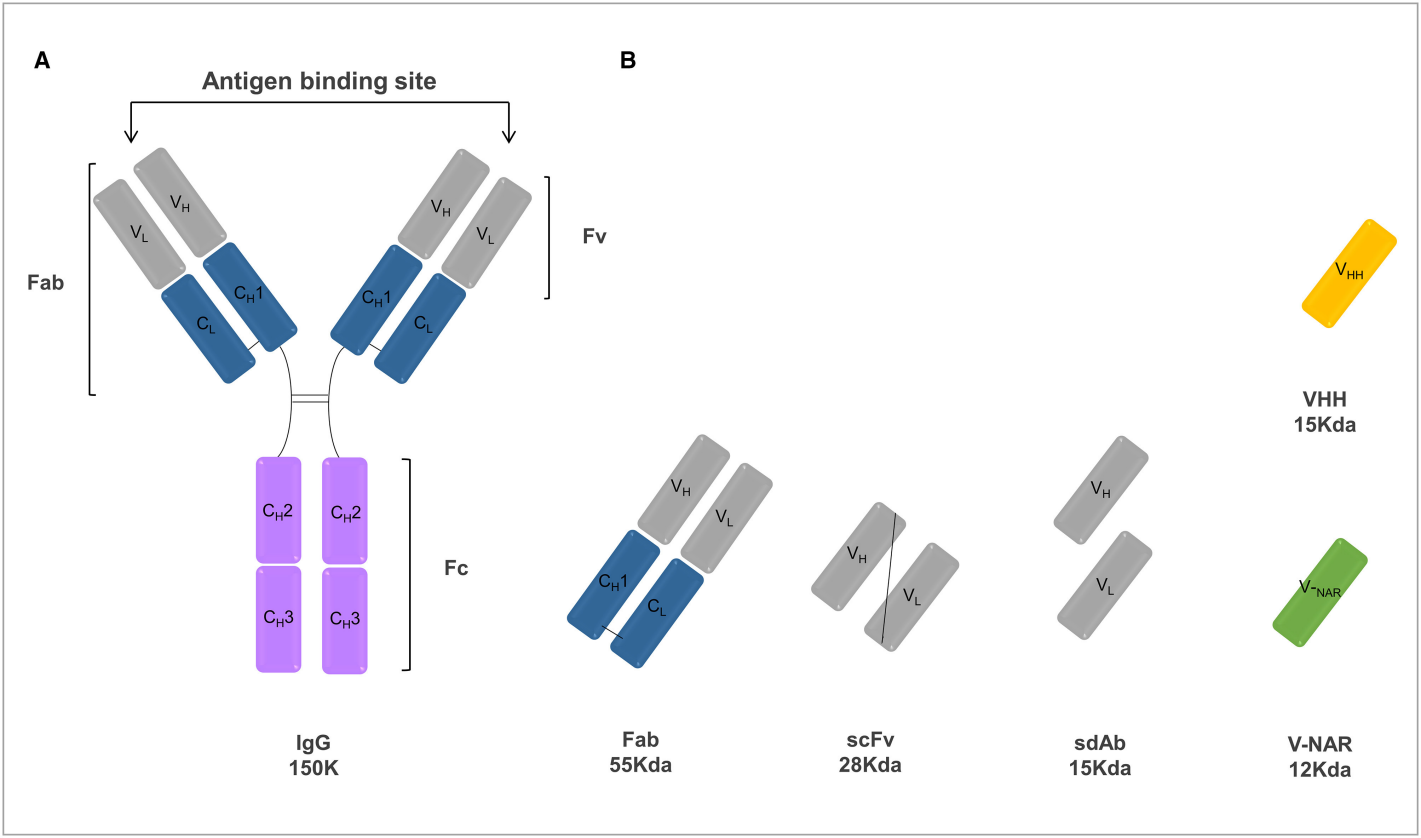
Schematic representation of various antibody formats including a conventional IgG antibody **(A)** and antibody fragments **(B)** of interest. **(A)** The basic unit of a conventional IgG antibody is a polypeptide consisting of a pair of identical heavy and light chains held together by disulfide bonds. Light chains are comprised of one constant domain (CL) and one variable domain (VL), whereas heavy chains are comprised of three constant domains (CH1, CH2, and CH3) and one variable domain (VH). The antigen-binding site is composed by the variable domains of both the heavy and light chains. In turn, the Fc constant region is responsible for the recruitment of the immune system effector functions. **(B)** Antibody fragments that can be engineered from a conventional IgG include: antigen-binding fragment (Fab), single-chain Fv fragment (scFv), heavy and light single domains antibodies (sdAbs) and natural camelid variable domain (VHH) and shark variable domains (V-NAR).

In cancer therapy, the main purpose of antibody treatment is to promote the direct or indirect death of cancer cells and a number of strategies have been successfully employed. MAbs can bind to target cancer cells and directly promote signaling-induced death or can mediate an anti-tumor immune response by promoting antibody-dependent cellular cytotoxicity (ADCC) and inducing complement-dependent cytotoxicity (CDC) (91). In the case of ADCC responses, mAbs bind to target tumor cells while the mAb Fc region engage with the FcγRs on the surface of effector cells, including natural killer cells and macrophages. These immune cells cause phagocytosis, apoptosis or lysis of the target cells. In CDC responses, mAbs promote directly target cell death through the development of a complement cascade membrane attack complex. Furthermore, mAb-based therapies can also block growth-promoting pathways, such as angiogenesis or can directly regulate the anti-tumoral activity of adaptive immune cells by blocking inhibitory signals responsible for limiting T cell activation (92). Most marketed mAbs consist of a full-length IgG molecule. By providing a long half-life and effector functions, these molecules have been presenting a quite successful application in therapeutics. However, this conventional antibody format present some drawbacks that limit their clinical use and there is a range of therapeutic applications in which other antibody formats may be more appropriate. To address these major issues, smaller antibody scaffolds such as the Fab or the single chain variable fragment (scFv) or single domain antibody are emerging as alternative therapeutic agents (93) (Figure 2).

MAbs are the most commonly used and approved cancer immunotherapy method in clinical practice (94). The use of an antibody targeting the human surface antigen CD20 (Rituximab®), expressed on B-lymphocytes has revolutionized the treatment of B-cell lymphoma (51, 52). Rituximab is a chimeric antibody and was the first US Food and Drug Administration (FDA) approved mAb for the treatment of human cancer, being used for the treatment of most B-cell NHL and subtypes of acute lymphocytic leukemia (95–97). This immunotherapy provided significant enhancements in the efficacy of treatment vs. existing non-mAb therapies, increasing the rate of durable remissions from 30 to 60% (51).

Even though immunotherapy has a crucial role in the treatment of B-cell malignancies in humans, its role in canine lymphoma remains limited. Immunohistochemistry using mAbs that recognize the CD20 intracellular domains demonstrated the presence of CD20 in canine lymphoma tissue samples (98, 99). However, Rituximab® and other anti-human and anti-mouse antibodies that recognize the CD20 extracellular domains, failed to bind to canine CD20, even though the reported epitopes are conserved between human and canine CD20 (100). For that reason, it is evident that technology to speciate antibodies is essential when developing similar passive immunotherapy strategies for canine cancer patients.

Interestingly, in 1992, prior to FDA approval of Rituximab, the United States Department of Agriculture (USDA) approved the licensing of mAb 231 for use in cNHL. mAb 231 consists of a murine-derived mAb that showed both *in vitro* (64) and *in vivo* activity and served as adjuvant therapy following remission induction with chemotherapy (65, 66, 81). Unfortunately, subsequent clinical trials failed to confirm the initial study results and the antibody epitope was never identified, which culminated in its commercial suspension (65).

Since then, driven by the great potential of the canine lymphoma model for immunotherapeutic approaches, academic research groups and industry began exploiting the dual benefit approach of comparative medicine.

One of the first examples was a pilot study that aimed to assess the suitability of the canine lymphoma model to evaluate endpoints with clinical relevance of anti-HLA-DR mAb treatment before proceeding to an extensive trial in pet dogs, and eventually human research. *In vitro* studies revealed that L243, a murine IgG1 anti-HLA-DR, binds to canine healthy lymphocytes and lymphoma cells, inducing apoptosis in cNHL cells. In turn, *in vivo* studies confirmed the L243 treatment safety in healthy dogs and dogs with lymphoma and its binding activity to lymphoma affected lymph node samples. Preliminary data also showed that a subset of patients with advanced lymphoma achieved transient disease stabilization after L243 treatment (67). Furthermore, this work also reported that hL243γ4P (IMMU-114), a humanized IgG4 anti-HLA-DR, under preclinical evaluation for human trials, also bound to cNHL cells. Finally, the assessment of IMMU-114 treatment in healthy canine patients indicated a safety and pharmacokinetic profile similar to L243. Overall, these findings supported the use of cNHL in safety and efficacy studies of anti-HLA-DR mAbs for both veterinary and human medicine (67).

Advances in speciation technology has also led to several clinical trials in pet dogs since “caninization” of antibodies is crucial when approaching canine patients with cancer. With this in mind, research groups focused on the technique to generate caninized antibodies, which resulted in the development of a canine anti-EGFR (epidermal growth factor receptor) mAbs (101) and nowadays is also being offered as a service by companies (*Creative Biolabs*).

Considering the success achieved with Rituximab in human medicine, several studies also focused on developing canine anti-CD20 antibodies. An anti-canine CD20 mAb (6C8) that recognized the extracellular domain of canine CD20 and showed high-affinity binding to canine CD20 in solution and its native conformation on canine B-cells was developed. This mAb promoted phagocytosis of B-cell lymphoma cells by macrophages, but in its current framework did not induce direct cytotoxicity or CDC (51). In the same year, Rue et al. reported the development of an anti-canine CD20 antibody (1E4) and the generation of a canine chimeric molecule for therapeutic use. This clone bound a similar extracellular domain as rituximab, and flow cytometry analysis confirmed that 1E4-based chimeric versions were able to stain canine B cells and canine CD16a, a receptor that mediates ADCC responses. Moreover, the best chimeric mAb candidate depleted the number of circulating B cells in healthy beagles in an *in vivo* study. Though, the clinical efficacy in dogs with canine B cell lymphoma remains unknown (68). Likewise, a new anti-CD20 mAb (NCD1.2) that bound both human and canine CD20 has been developed, in order to strengthen human-canine comparative model. NCD1.2 bound to clinically derived canine cells including B-cells in peripheral blood and in different histologic types of B-cell lymphoma. Heavy chain and light chain genes from the NCD1.2 hybridomas were cloned and packaged as scFv into a phage-display library. Recombinant anti-CD20 scFv were identified and selected as a possible useful tool for evaluation in bioconjugate-directed anti-CD20 immunotherapies in comparative medicine (69). Although these works established several canine anti-CD20 mAbs candidates with high potential for therapeutic use, their clinical efficacy in dogs bearing B-cell lymphoma remains unknown.

A canine anti-CD20 mAb (AT-004) has been fully approved by USDA for clinical usage in dogs with B-cell Lymphoma and is currently being commercialized in the United States and Canada. Treatment with AT-004 (*Aratana Therapeutics*), an anti-canine CD20 was subject to a prospective randomized clinical trial and preliminary results suggested an improved median progression-free survival of dogs with B-cell lymphoma (102). Yet, these results were published in a conference abstract and peer-reviewed results are still lacking. Another work evaluated the combination of CD47-blockade with 1E4-cIgGB, a canine-specific antibody to CD20. Although 1E4-cIgGB could elicit an *in vivo* therapeutic response against canine lymphoma as a single agent, superior responses were observed when combined with agents targeting CD47, an immune checkpoint that enables the evasion of tumor cells to phagocytosis promoted by therapeutic antibodies, such as anti-CD20 mAbs. The combination of CD47-blocking therapies with 1E4-cIgGB resulted in synergic antitumoral effects *in vitro* and *in vivo*, eliciting cures in 100% of mice bearing canine lymphoma (45). However, there is no anti-CD20 antibody treatment for cNHL currently available. More recently a novel approach of developing an anti-canine CD20 monoclonal antibody using rats as a host species renewed hopes of finally obtaining an antibody-based therapy for cNHL. This work culminated in the generation of a mAb capable of inducing cell death of B cell lymphoma cell lines, however this mAb was incapable of eliciting CDC and ADCC responses. To tackle these limitations, this antibody was modified into a canine/rat anti-CD20 chimeric, which resulted in the alterations of its characteristics into a potent CDC and ADCC inducer. Furthermore, its defucosylation resulted in a 10-fold higher ADCC activity. The *in vivo* antitumor activity of this improved mAb version was assessed, revealing a tumoral growth inhibition in a cNHL xenograft mouse model and a peripheral B cell depletion in healthy beagles (70). Finally, AT-005 (*Aratana Therapeutics*), a caninized mAb targeting CD52 on T cells, has obtained conditional USDA approval for the treatment of T-cell lymphoma and is currently being evaluated in clinical trials (62).

The success of mAbs in human medicine strongly encourages veterinary medicine to develop similar therapeutics for our pets. Regardless of their potential, little speciated mAbs have been established for veterinary application and fewer were investigated in clinical trials enrolling companion animals. Nonetheless, the approval of the first mAb by the European Union Agency for the treatment of atopic dermatitis in dogs—Lokivetmat, a caninized, anti-canine IL-31 mAb (103), highlighted the impact that biological therapies may have in veterinary practice. In the oncology setting, mAbs have the capacity to treat a diversity of hematological and solid malignancies, do not need to be a personalized product and manufacturing methods are well-established, minimizing the cost associated limitation. Hence, mAb-based therapy is one of the most promising immunotherapy strategies in veterinary settings (63).

### Adoptive T-Cell Transfer

Adoptive cell therapy is a term that was first used to describe the infusion of lymphocytes to mediate rejection of organ allografts and to treat tumors (104). This immunotherapeutic option represents the most effective treatment for patients with metastatic melanoma inducing visible cancer regression in ~50% of patients. Adoptive cell therapy is also associated with clinical improvement in selected patients with post-transplant lymphoproliferative diseases caused by Epstein–Barr virus infection (105). More recently, gene transfer techniques developed in the 1990s allowed to convert normal lymphocytes into lymphocytes with anti-cancer activity by redirecting the specificity of T cells with the use of T-cell receptors or chimeric antigen receptor (CARs). CARs are engineered receptors that graft a defined specificity onto an immune effector cell, typically a T cell, resulting in the augment of T-cell function (104). This innovation represented a possibility of extending adoptive cell immunotherapy to patients with a large diversity of cancer types (105). In humans, treatment of advanced B-cell leukemia or lymphoma using CAR T-cells has demonstrated promising clinical responses, resulting in the approval of two autologous CAR T-cell therapies (Kymriah™ and Yescarta™) by the FDA (106, 107). These therapies are both genetically modified autologous T cells expressing a CD19-specific CAR, lysing CD19-positive targets (107).

By displaying an intact immune response and genetic similarities to humans, dogs may potentially inform the development of the later-stages of human clinical trials, while studying the use of adoptive cell therapy in veterinary malignancies, including hematologic neoplasias (71, 72). In fact, there is evidence that canine cancer, and specifically cNHL, respond to cell-based immunotherapy. Half a century ago, the Fred Hutchinson Cancer Center established hematopoietic cell transplantation for canine lymphoma (108). At first, the therapeutic value of this practice was solely associated with the administration of high-dose chemotherapy and radiation prior to the transplant. Yet, a larger retrospective study confirmed that, despite the use of the same chemotherapy and radiation protocols, dogs that received an allogeneic transplant from a littermate exhibited a significantly lower relapse rate, in contrast to dogs that received their own (autologous) bone marrow stem cells. This effect was later known as the “*graft vs. leukemia/tumor effect*” and is mainly promoted by activated allogeneic T cells that recognize and react to antigen differences, and therefore also attack residual tumor cells (109).

Since then, few studies have focused on the scientific and clinical investigation of cell-based immunotherapies for canine patients. O'Connor et al. conducted a clinical trial to test non-specific autologous T cells isolated from dogs with NHL and expanded *ex vivo* using a novel artificial antigen presenting cell protocol (71, 72). Infused cells were detected in the blood for longer than 49 days and trafficked to secondary lymphoid organs, confirming the safety of adoptive transfer of autologous T cells in dogs. Furthermore, this adoptive immunotherapy demonstrated to be viable and effective in improving first remission and overall survival periods in dogs with multicentric lymphoma (71, 72).

Notably, a few biotech companies have emerged in the area of autologous T-cell based therapy for veterinary medicine. One example is *Aurelius BioTherapeutics* that provides a service that expands for 2–3 weeks autologous lymphocytes collected from dogs with canine lymphoma, in order to increase T cell numbers exponentially and to activate them to be responsive to antigens presented by the tumor cells before reinfusion. However, the methods used for the activation and expansion of dog's immune cells and the clinical benefit of this therapy are not disclosed. In turn, *Elias Animal Health* included a vaccination procedure prior to cell collection, aiming to improve cancer-cell specificity of their autologous T-cell therapy. The vaccine is obtained from the excised tumor material and is given through an intradermal route. Additionally, a brief cycle of chemotherapy may be administered prior to the infusion, which has shown to result in better acceptance of the lymphocyte therapy in humans. The preliminary results revealed that overall survival may be prolonged with this adoptive cell-based therapy, indicating that this immunotherapy prompts an antitumor vaccine-like effect that extends canine patients' lives, even when the disease is not fully eradicated. The holding company is pursuing regulatory approval, which would qualify it as the first approved and commercialized cell therapy for dogs (106).

**Figure 3:**
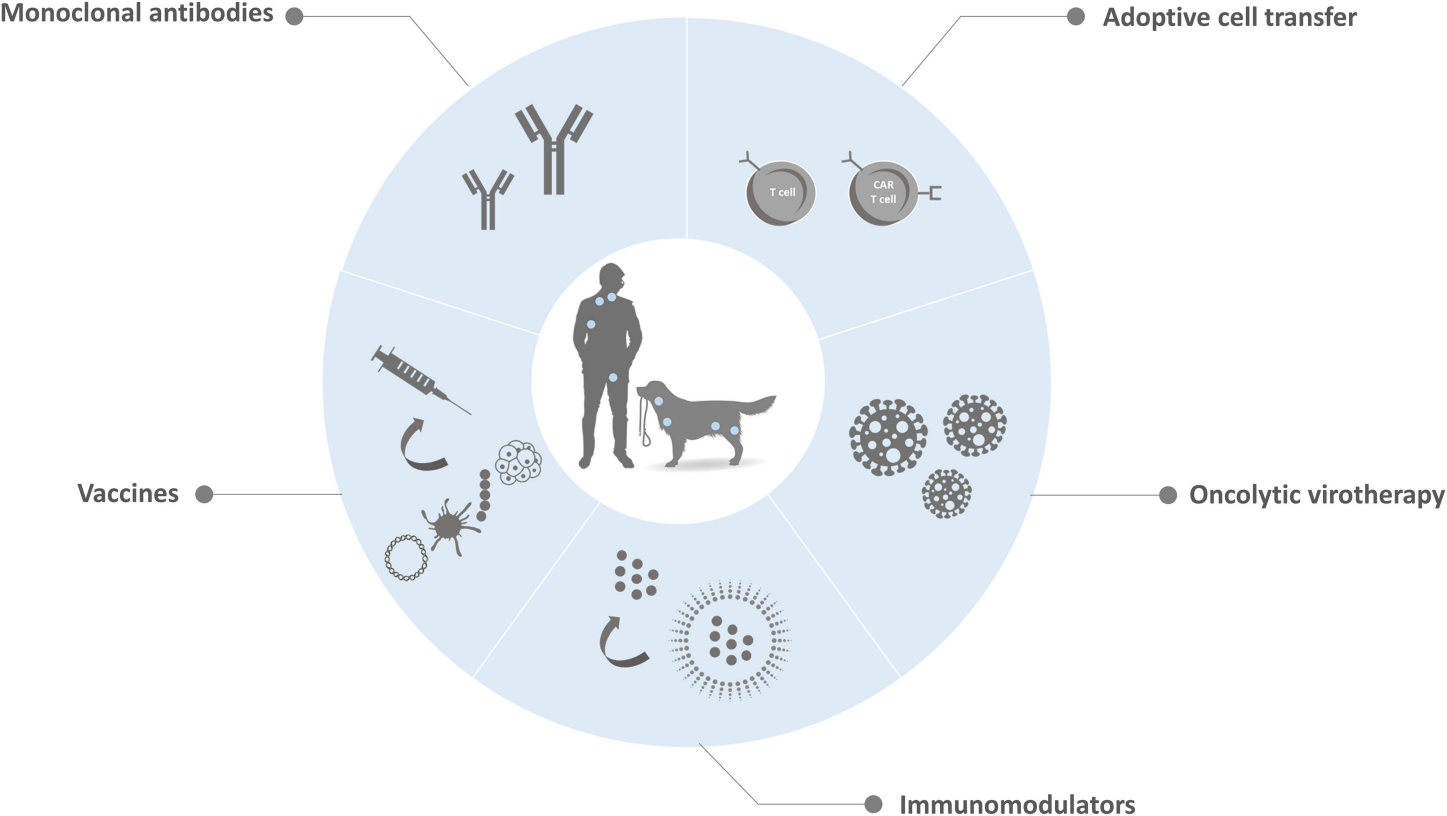
CAR-T cells therapy. The basic procedures for CAR-T cell therapy start with the collection and extraction of T cells from the pet's peripheral blood. The T cells are then genetically engineered *in vitro* to express chimeric antigen receptors (CARs) that can recognize specific tumor-associated antigens and activate self-proliferation and cytotoxicity. Finally, CAR-T cells are .

More recently, researchers have started to explore chimeric antigen receptor T-lymphocytes (CAR-T) cell therapy for dogs (Figure 3). CARs engineering consists of modifying T-cells to express artificial receptors formed by a tumor-antigen specific scFv linked to an intracellular signaling domain and co-stimulatory molecules. Because CARs work in a MHC independent manner, antigen presentation do not rely on patient antigen presenting cells. Moreover, CARs do not have to be syngeneic to the patient immune system (63). Canine T cells expressing a HER2 (human epidermal growth factor receptor 2)-specific CAR have been produced and showed anti-tumoral activity *in vitro* against canine osteosarcoma cells expressing HER2 (110). This work proved that a successful *ex vivo* expansion of HER2-CAR specific T-lymphocytes is possible. Yet, no canine patients have been treated. Ongoing studies aim to develop a canine CAR-T cells for the treatment of B-cell lymphomas and other malignancies (63). Importantly, protocols for the propagation of CD20 CAR-T cells have been reported (73, 111). Researchers transfected the CD20 CAR into the expanded T-cells using electroporation of CAR mRNA. Unfortunately, even though this strategy allows to avert using retro or lentivirus, mRNA transfection results in variable efficiency and transient transcriptional activity that ceases following 24 to 48 h. It was reported the treatment of one dog diagnosed with lymphoma with these transfected T-cells, however it only presented a short-term partial response (73, 106). This limited clinical response can be due to the inability of these transfected cells to expand *in vivo*, considering that human studies demonstrated that *in vivo* expansion is a requirement for durable responses. Furthermore, this treatment protocol did not include chemotherapy sessions prior to the CAR-T cells infusion, a common practice used in the human treatment to deplete inhibitory immune cells that has shown to potentiate clinical efficacy. In the case of dogs, the addition of this procedure could also minimize the risk of triggering a canine anti-mouse antibody immune response, considering that most scFvs derived from murine mAbs, thereby increasing the risk for an anti-CAR T cell immune response. To conclude, reported data proved the feasibility of generating canine CAR-T cells, however the necessary logistics and expenses are expected to be considerable.

### Oncolytic Virotherapy

Oncolytic virotherapy is a new concept of immunotherapy recently introduced that involves the replication-competent virus in the elimination of cancer. By infecting tumor cells, oncolytic virotherapy can stimulate *de novo* or enhance pre-existing native immune response. The majority of developed oncolytic virus are genetically altered to promote tumor tropism while reduce virulence against healthy host cells. Thereby, oncolytic virotherapy have the ability to promote a proinflammatory environment by improving antigen release/recognition and promoting immune activation, while reverting immunosuppression of tumor cells and improving the efficacy of other forms of immunotherapy (112, 113). Although several oncolytic virotherapies are being developed in preclinical and clinical settings, currently the only oncolytic viral therapy approved by FDA is talimogene laherparepvec (T-Vec or Imlygic) for advanced melanoma (114). In veterinary medicine, several studies evaluated natural and genetically modified oncolytic viruses for dogs diagnosed with cancer, showing some encouraging results. However, the majority of the developed research work focused on *in vitro* results, with a few reporting *in vivo* studies, of which most were isolated clinical case reports (115).

Regarding cNHL, a study reported that a recombinant strain of the canine distemper virus (CDV)—pCDVeGFPΔN—was capable of infecting cNHL cell lines *in vitro*, inducing significant apoptotic cell death. The pCDVeGFPΔN strain also efficiently infected primary canine B and T-cell lymphoma cells, though its oncolytic efficacy was not proved (74). Another work evaluated the anti-tumoral effect of CDV infection using an attenuated strain in seven dogs with naturally occurring lymphoma. For this purpose, single or multiple doses of the virus were injected intratumorally. This study reported low toxicity with a severe fibrotic reaction in the injection site. Immunohistochemistry analysis revealed a variable positive detection of CDV antigen in treated lymph nodes, while co-culturing enabled virus isolation from treated lymph nodes, but not from distant nodes or from peripheral blood mononuclear cells (PBMCs). Furthermore, this treatment promoted a strong anti-CDV antibody response (75). However, one of the major drawbacks of this immunotherapy is that CDV belongs to the regular vaccination schedule in dogs and pre-existing antibodies can limit its efficacy (116). Another group explored the oncolytic properties of a vaccine strain of Newcastle disease virus, an attenuated lentogenic strain presenting low virulence, on a human large B-cell lymphoma cell line and on primary canine B-cell lymphoma cells. The group used as controls healthy PBMCs from humans and dogs. Newcastle disease virus infection decreased cell viability in both human and dog lymphoma cells when compared to untreated controls, with minimal tropism toward healthy PBMCs. In the same work the authors reported the viral biodistribution in a canine patient diagnosed with T-cell lymphoma, 24 h following the virus intravenous injection. Immunohistochemistry and endpoint PCR demonstrated viral dissemination in the salivary gland, kidney, stomach and lung, but not in tumor samples, with no abnormal findings on the histopathological evaluation (76). Curiously, a complete and long-term clinical response was reported in a dog diagnosed with lymphoma resistant to chemotherapy (76, 77). Although these preliminary data revealed that Newcastle disease virus could represent a promising oncolytic virotherapy, future studies are required to determinate the best therapeutic regimen and define the proper safety protocol (117).

One of the oncolytic virotherapies that has gathered most interest amongst the scientific community, due to the promising results obtained in multiple phase I and II clinical trials, is the dearing strain of Reovirus (Reolysin®, from OncolyticsTM Biotech Inc., Calgary, AB, Canada) (118). In dogs, Reolysin® showed promising *in vitro* results for the treatment of a variety of malignancies, such as mastocytoma, lymphoma, mammary gland tumors and melanoma. In fact, *in vitro* studies showed apoptosis induction and a significant cell viability reduction in both T and B-cell lymphoma. Furthermore, a mouse xenograft model of canine T-cell lymphoma treated via intratumoral injection revealed significant tumor growth inhibition, compared to the control group treated with reovirus inactivated by ultraviolet (78). Notably, the safety profile of Reolysin® was proven in a clinical trial enrolling dogs with advanced cancer, including mastocytoma, lymphoma, oral melanoma and soft tissue sarcoma. In this work, dogs received virotherapy by intratumoral injection or intravenous injection daily for 5 days, during one or several treatment cycles. Live virus was only detected in the serum of one dog in the first chemotherapy cycle, but not in the subsequent treatment cycles. While all dogs exhibited an increase in the titer of anti-reovirus neutralizing antibodies, tumor volume reduction was observed in five dogs and six dogs presented alleviation of clinical manifestations. Furthermore, a subset of dogs revealed a good safety profile, as well as clinical response. Taking into account the experience gathered in human medicine, the combination of this immunotherapy with conventional therapies such as chemotherapy, radiotherapy, or other could be investigated in dogs (79).

Overall, these studies provide preliminary results that support the development of oncolytic virotherapy as canine cancer therapy to benefit pets and pet-owners (115).

### Immunomodulators

Cytokine therapy aims to enhance immune responses and tumor control in a variety of spontaneous oncologic diseases. In human medicine, modest success has been obtained with a low-dose IL-2 therapy delivered subcutaneously, with few side effects (119–124). Additionally, subcutaneous GM-CSF (Granulocyte-macrophage colony-stimulating factor) therapy boosts cell-mediated immune responses and improves anti-idiotype vaccines efficacy in human lymphoma (125). In canine patients, IL-2 delivered subcutaneously, intralesionally, by inhalation and via liposome-DNA complexes encoding IL-2 gene, as a monotherapy or in combination with other modalities, promoted regression in dogs with oral melanoma, soft tissue sarcoma, squamous cell carcinoma and pulmonary metastases from osteosarcoma (126–131). Likewise, in dogs with oral melanoma, combination therapy including GM-CSF delivered intralesionally, either via liposome–DNA complexes or via GM-CSF secreting transgenic xenogeneic cells, resulted in regression (126, 132). Through the Comparative Oncology Trials Consortium, a Phase I safety/dose escalation study of human IL12 administered subcutaneously to dogs with melanoma was conducted. Data gathered from this study and other preclinical data allowed to inform the design of a Phase I clinical trial of IL12 in human cancer patients (133).

A phase I study enrolling 15 dogs with B-cell lymphoma tested a therapy with a combination of autologous tumor antigen-coated microbeads (large multivalent immunogen—LMI) with cytokine therapy including IL-2 and GM-CSF, following induction of remission with conventional chemotherapy. Results demonstrated no significant toxicity, no adverse effects in disease-free interval and half of the animals presented quantifiable delayed-type hypersensitivity reactions to intradermal LMI, suggestive of a specific cell-mediated immune response (80).

Although these studies show that human cytokines can be effectively used in dogs, the often-needed higher doses and the immunogenicity that they generate, limits their use. Nonetheless, the development of canine IL-15 has led to a renewed interest in cytokine therapy as an immunotherapy strategy for veterinary settings (134).

### Vaccines

Therapeutic vaccines represent a viable and attractive cancer immunotherapy strategy that aim to treat late stage disease by stimulating a patient's own immune system against cancer cells (135).

Several attempts to use vaccines as a treatment for cNHL have been made. In the initial studies, Freund's adjuvant was added to lymphoma cell extracts lysates and used as a cancer vaccine strategy. Despite de fact that these early studies reported some treatment benefit (136), this was later attributed to the use of the Freund's adjuvant (137).

Later, Jeglum et al. described the use of an autologous tumor vaccine administrated via intralymphatic injection following remission induction with chemotherapy. However, results using this strategy have been conflicting (81–83).

In a clinical trial, autologous CD40-activated B-cells loaded with total RNA from autologous lymphoma cells were administered to 19 dogs with NHL as an adjuvant, following induction of a complete response with chemotherapy. Vaccination promoted an anti-tumor response and increased a lasting second remission rate, however median time to disease progression and overall survival did not show differences between groups (84).

Moreover, a new approach targeting canine telomerase reverse transcriptase using a genetic vaccine, Tel-eVax, is reported. As telomerase confers immortality to cells, telomerase reverse transcriptase is overexpressed in cancer cell lines and in several tumors and undetectable in the majority of normal tissues, establishing a possible target for translational cancer immunotherapy. A DNA-vaccine targeting canine telomerase reverse transcriptase was able to prompt an immune response against telomerase in dogs diagnosed with multicentric lymphoma, and conventional chemotherapy seems not to alter the immunotherapy effects (85). The combination of this vaccine with chemotherapy using the cyclophosphamide, vincristine and prednisolone protocol resulted in a durable immune response, as well as prolonged survival in dogs with B-cell lymphoma (86). On other clinical study including 17 pet dogs, Tel-eVax in association with CHOP chemotherapy demonstrated to be safe and immunogenic and presented a significant impact on DLBCL canine patients' survival. Antibody response induced by Tel-eVax against telomerase reverse transcriptase (TERT) protein was also evaluated considering the potential that these anti-TERT antibodies may possess as surrogate biomarkers of the immune response in vaccinated dogs. Curiously, most dogs developed a low but detectable seroconversion against the N-terminal of TERT protein (87).

More recently, an autologous vaccine APAVAC®, comprised of hydroxylapatite ceramic powder with autologous heat shock proteins (HSP) purified from affected lymph node biopsy is currently available (88). HSPs resultant from tumor cells, including gp96, hsp90, hsp70, calreticulin, hsp110, and hsp170, present strong immunogenicity. Furthermore, the chaperone function of HSPs allows their combination with immunogenic tumor specific peptides (HSPPC), exposing the host to a large repertoire of tumor associated antigens for immunization. In addition, hydroxylapatite vehicles and HSPPCs functions as an adjuvant. In order to reproduce the tumor heterogeneity, each vaccine is produced for each dog. Vaccination protocol consists of four administrations within 4 weeks followed by one injection a month for 4 months in combination with dose-intense chemotherapy. In an initial phase, preliminary results showed that the administration of this autologous vaccine is effective in prolonging overall survival and the time to progression in dogs with DLBCL and multicentric indolent B-cell neoplasia, without increasing treatment toxicity (88, 89). Following, to better characterize the safety and efficacy of APAVAC®, and to find the best candidates for immunotherapy, a larger retrospective study was conducted, which included all dogs treated with chemo-immunotherapy to date. Overall, compared to dogs treated with chemotherapy only, dogs receiving the chemo-immunotherapy protocol survived significantly longer, regardless of histotype and evaluated prognostic factors. The study also confirmed the excellent tolerability of the vaccine in dogs diagnosed with B-cell lymphomas (90). Unfortunately, until now there is no information regarding the chemo-immunotherapy treatment response in T-cell lymphoma dogs.

Altogether these works clearly demonstrate the potential of the cNHL model to advance cancer vaccine strategies research to treat lymphoma both in humans and dogs.

### Immune Checkpoint Blockade

Immune checkpoint inhibitors, such as those targeting CTLA-4 and the PD-1 (programmed-death 1)/PD-L1 (PD ligand 1) axis, have shown unprecedented and durable clinical effect in a wide range of malignancies and are rapidly transforming the practice of medical oncology in humans (138).

Tumor cells can successfully evade immunosurveillance and progress through different mechanisms, including activation of immune checkpoint pathways that hinder antitumor immune responses. By interrupting co-inhibitory signaling pathways, immune checkpoint inhibitors reestablish antitumor immune responses and promote immune-mediated elimination of malignant cells (139). Hematologic malignancies such as lymphoma are likely targets for this type of treatment. Several clinical trials of checkpoint blockade have been performed in hematological malignancies, with promising preliminary results, suggesting the therapeutic benefit of this approach. These results were specially promising regarding PD-1 blockade in Hodgkin lymphoma (140). To date, there are currently seven approved immune checkpoint inhibitors for the treatment of various cancers in human medicine.

Clinical trials using checkpoint inhibitors for the treatment of cNHL have yet to be conducted. Nevertheless, expression of canine PD-L1 has been reported on a variety of canine tumor types, including mastocytoma, melanoma and renal cell carcinoma (141). A preliminary study suggests that anti-PD-L1 might play a significant role in the treatment of dogs with tumors expressing PD-L1, by demonstrating that treatment of canine tumor infiltrating lymphocytes with this molecule improved interferon-γ production (141). It was recently reported that PD-L1 is elevated in canine B cell lymphomas compared to normal B cells. Tumor cells from T-cell cNHL and healthy canine patients both showed low to negative expression of PD-1 and PD-L1. In addition, tumor infiltrating lymphocytes from both B-cell and T-cell lymphoma cells presented an increased expression of both PD-1 and PD-L1 expression compared to B and T cells from lymph nodes of healthy animals. *In vitro*, chemotherapy-resistant canine B-cell and T-cell lymphoma cell lines exhibited increases in both PD-1 and PD-L1 expression, compared to non-chemotherapy selected tumor cells (142). In line with this, a panel of 5 canine PD-1/PD-L1 mAbs were generated and are being studied for *in vitro* activity in T cell assays (143). Moreover, the immunomodulatory effects of c4G12, a canine-chimerised anti-PD-L1 mAb, were evaluated *in vitro*, demonstrating significantly enhanced cytokine production and proliferation of dog PBMCs. Then, a pilot clinical study was performed on seven dogs with oral malignant melanoma and two with undifferentiated sarcoma, revealing that this antibody can be a safe and effective treatment option for canine cancers (144).

Importantly, canine CTLA-4 (cytotoxic T-lymphocyte associated protein 4) has also been described and cloned (145). An agonistic recombinant canine CTLA has been efficiently used to promote tolerance in a transplant model (146), suggesting that the mechanism of action of CTLA-4 in dogs is similar to humans and that CTLA-4 checkpoint blockade could represent a novel immunotherapy for canine cancer. Importantly, Tagawa et al. (147) demonstrated an up-regulation expression of CTLA-4 on CD4+ T cells from peripheral blood obtained from dogs with B cell high grade lymphoma. CTLA-4 expression on T cells was also associated with a poor prognosis.

With the development of new checkpoint molecule targeted drugs for dogs, multiple opportunities emerge in which the dog model may provide relevant clinical information, especially regarding the rational combination of immunotherapies, including checkpoint inhibitors.

## Discussion

The current landscape of cancer research is facing a profound transformation with the introduction of immune-oncology as the fourth pillar for cancer therapy. Not only have immunotherapies resulted in unprecedented clinical responses, rapid drug development and several first-in-class approvals from the FDA in the past few years, but the advent of such innovative therapies is also revolutionizing treatment paradigms and algorithms in current oncology and hemato-oncology practice (148). As a result, clinical and translational research need to adapt to a rapidly changing scenario to effectively translate novel concepts into sustainable and accessible therapeutic options for cancer patients (149). The complexities and challenges of the new era of immune-oncology strongly emphasize the need to identify new strategies, models and paths to develop fast, successful, and cost-effective therapies (13, 149). The inclusion of a canine model in the drug development path of cancer immunotherapies is being widely recognized as a valid solution to overcome several hurdles associated with conventional preclinical models (150). Dogs with naturally occurring tumors are highly translational models that represent an opportunity to investigate the clinical potential of novel immunotherapies in a comprehensive manner. By complementing murine studies and human clinical trials, dogs allow monitoring the “scaling up” effects of a therapeutic approach that depends on complex interactions between tumor and immune cells, while assessing long-term efficacy and toxicity (15). Taken together, these features may allow the establishment of solid foundations to rapidly translate the results obtained from canine patients to human patient management, with benefits for both species (151).

Importantly, the benefits of these collaborative studies can more easily translate into clinical success in emerging technologies, such as immune checkpoint inhibitors and CAR T cells therapy, where the rapid pace of its clinical applicability is proving critical challenges. In fact, a lot remains to be understood about patient selection, delivery, and off-target effects of emerging immunotherapy used alone or in combination. While clinicians have learned during the last decades to deal with clinical responses and toxicities related to the use of antibodies in cancer therapy, emerging therapies, such as those mentioned, are much less familiar to oncologists. Therefore, cancer research needs to develop better predictive clinical models to make these emerging immunotherapies universally available to those patients with cancer who need immune intervention in addition to other therapies (152).

However, the implementation of such canine clinical trials is far from being an easy quest. It requires multiple organized efforts to validate the canine model, which still lacks a thorough characterization of the canine immune system and its effector cells and molecules, the evaluation of common tumor epitopes, the development of canine-specific/cross-reactive agents and the establishment of preclinical models for veterinary oncological settings (62, 153, 154). Furthermore, this also requires veterinary scientific community to join forces to implement diagnosis, staging and treatment response assessment optimization and standardization, to perform large and organized clinical trials and to achieve conformity when analyzing data (26).

Regardless of the challenges that implementing immunotherapies for cNHL lymphoma may pose, cNHL treatment is facing a paradigm shift. With several new immunotherapies emerging, it is expected that in the nearby future, immunotherapy will become a valid therapeutic tool, along with chemotherapy, radiotherapy and surgery. Furthermore, these advances also provide an integrated drug discovery platform that maximize interdisciplinary cooperation and leverage commonalities across humans and dogs, for the development of novel immunotherapies against NHL, benefiting both species.

## Author Contributions

JD: writing—original draft preparation and visualization. AA: visualization and writing—review and editing. SA, SG, LT, and FA-d-S: writing—reviewing and editing. All authors contributed to the article and approved the submitted version.

## Conflict of Interest

The authors declare that the research was conducted in the absence of any commercial or financial relationships that could be construed as a potential conflict of interest.

## Publisher's Note

All claims expressed in this article are solely those of the authors and do not necessarily represent those of their affiliated organizations, or those of the publisher, the editors and the reviewers. Any product that may be evaluated in this article, or claim that may be made by its manufacturer, is not guaranteed or endorsed by the publisher.

## Abbreviations

ADCC: antibody-dependent cellular cytotoxicity
CAR: chimeric antigen receptor
CAR-T: chimeric antigen receptor T-lymphocytes
CDC: complement dependent cytotoxicity
CDV: canine distemper virus
CHOP, cyclophosphamide, doxorubicin: vincristine and prednisolone
cNHL: canine lymphoma
CTLA-4: cytotoxic T-lymphocyte associated protein 4
DLBCL: diffuse large B-cell lymphoma
EGFR: epidermal growth factor receptor
FDA: US Food and Drug Administration
GM-CSF: Granulocyte-macrophage colony-stimulating factor
HER2: human epidermal growth factor receptor 2
hNHL: human non-Hodgkin lymphoma
HSP: heat shock proteins
HSPPC: immunogenic tumor specific peptides
LMI: large multivalent immunogen
mAbs: monoclonal antibodies
NHL: non-Hodgkin lymphoma
PBMC: peripheral blood mononuclear cells
PD-1: programmed-death 1
PD-L1: PD ligand 1
scFv: single chain variable fragment
TERT: telomerase reverse transcriptase
USDA: United States Department of Agriculture.
